# VHL tumor suppressor as a novel potential candidate biomarker in papillary thyroid carcinoma

**DOI:** 10.17305/bjbms.2022.7850

**Published:** 2023-01-06

**Authors:** Lidija Todorović, Boban Stanojević

**Affiliations:** 1 Laboratory for Radiobiology and Molecular Genetics, Vinča Institute of Nuclear Sciences, National Institute of the Republic of Serbia, University of Belgrade, Belgrade, Serbia; 2 Department of Haematological Medicine, Division of Cancer Studies, Leukemia and Stem Cell Biology Team, King’s College London, London, UK; 3 Virocell Biologics, Department of Cell and Gene Therapy, Great Ormond Street Hospital for Children, Zayed Centre for Research into Rare Disease in Children, London, UK

**Keywords:** Papillary thyroid cancer, papillary thyroid carcinoma (PTC), Von Hippel–Lindau (VHL), biomarker, risk stratification, tumor suppressor

## Abstract

Papillary thyroid carcinoma (PTC) is the most common type of endocrine cancer, with an increasing incidence worldwide. The treatment of PTC is currently the subject of clinical controversy, making it critically important to identify molecular markers that would help improve the risk stratification of PTC patients and optimize the therapeutic approach. The Von Hippel–Lindau (*VHL*) tumor suppressor gene has been implicated in tumorigenesis of various types of carcinoma and linked with their aggressive biological behavior. The role of VHL in the origin and development of PTC have only recently begun to be revealed. In this narrative review, we attempt to summarize the existing knowledge that implicates VHL in PTC pathogenesis and to outline its potential significance as a candidate molecular biomarker for the grouping of PTC patients into high and low risk groups.

## Introduction

Thyroid cancer represents the most common malignancy present in the endocrine organs. Over many decades its incidence has increased worldwide, generating an additional burden on healthcare systems. Papillary thyroid carcinoma (PTC) alone makes up over 80% of all thyroid cancers and for about 95% of the increased incidence worldwide [1, 2].

Molecular biomarker analysis is a significant addition to the traditional pathological evaluation of carcinoma and represents a valuable tool for improving diagnosis and refining clinical management. A number of genetic mutations and other molecular alterations can be detected in fine-needle aspiration biopsies (FNAB) of thyroid nodules and can be of help in diagnosing cancer in a patient and treating patients with thyroid nodules. The American Thyroid Association identified mutations in seven genes and recommended, in its guidelines, that a seven-gene molecular biomarker panel of genetic mutations and rearrangement be set up and tested in FNAB samples [3–5]. This panel consists of *BRAF^V600E^*, three isoforms of *RAS* point mutations and translocations of *PAX8/PPARγ* and *RET/PTC* genes. The identification of any of these genetic changes in a thyroid nodule would represent a higher risk of malignancy, which is particularly important for a high number of patients who present with non-specific FNAB cytology. However, there is mounting evidence that the seven-gene MT test shows wide variation, ranging from 44% to 100% [3–6].

Although important advances have been made in the identification of specific genetic alterations and the fundamental role of several signaling pathways in thyroid cancer pathogenesis, 30%–35% of differentiated thyroid carcinomas, including PTC, lack any of these alterations [7]. Therefore, a pressing need to find new and more relevant molecular biomarkers to aid early diagnosis of PTC in order to rule in the malignancy for cytology indeterminate nodules exists.

Risk stratification of diagnosed patients represents another major issue in PTC management. The key outcome to predict is persistent/recurrent disease since, for the majority of PTC cases, the mortality risk is low. PTC tumors are slow growing, so patients usually have an excellent prognosis. However, reports by several groups show that 20%–30% of cases develop recurrence [8–10]. In rare cases, PTC may progress to an undifferentiated thyroid tumor, or the tumor may lose all the differentiation markers and transform into an anaplastic thyroid carcinoma (ATC), a very aggressive form of tumor which is characterized by poor prognosis with very low survival rates [10, 11]. Currently, the prognosis of PTC is essentially based on clinical and pathological factors; among them are: the patient’s age, tumor size, nodal and distant metastases, extrathyroid spread, and histotype [10, 12]. More recent studies suggest that mutational and expressional molecular alterations could be a significant addition to conventional evaluation and a critical addition toward personalized treatment of PTC patients [13–16].

Von Hippel–Lindau (*VHL*) is a tumor suppressor gene, and loss of its suppressor function is seen in heritable cancers linked with VHL syndrome as well as in some sporadic cancers [17, 18]. A number of reports have shown that VHL protein plays a critical role in oxygen signal transduction, but there is growing evidence to suggest that the function of VHL is likely to extend beyond this and that the loss of its function may result in deregulation of several signaling pathways that have critical roles in biological processes, notably cell proliferation, survival, invasion, and metastasis [19, 20]. In recent years, several lines of evidence, including our two studies, suggest that the *VHL* gene plays an important role in the development and progression of PTC. This evidence is the focus of the present narrative review.

## Application scope and effect of existing molecular markers of PTC

RET-RAS-RAF-MAPK pathway is commonly found activated in PTC. It promotes cell growth, differentiation, proliferation, and survival. The *RET/PTC* gene rearrangements, *RAS*-family genes, and point mutations in the *BRAF* are the most usual genetic changes that activate this pathway in PTC. These genetic mutations are responsible for up to 70% of all PTCs. These genes work independently of each other since each can result in uncontrolled downstream effects. They can therefore be characterized as virtually mutually exclusive [7, 21, 22].

*RET* gene rearrangements, known as *RET/PTC*, are identified in about 20% of adults with PTC, 40%–70% of children and adolescents with sporadic PTC, and in 50%–86% of irradiated patients [23]. *RET/PTC1* and *RET/PTC3* are the most common *RET/PTC* rearrangements found in PTC. Some pathological features of PTC, e.g., large tumor size and lymph node involvement, are found to correlate with *RET/PTC* rearrangements [24].

The most common mutation found in PTC in adults is the thymidine to adenine conversion at nucleotide 1799 of exon 15 of the *BRAF* gene. This has a frequency of 29%–83%, and results in a valine to glutamic acid substitution at amino acid residue 600 (BRAF^V600E^) [22, 25]. To a lesser degree, *BRAF^V600E^* is also detected in poorly differentiated thyroid carcinoma and ATC arising from PTC. This accords with the results in model cells, which suggest that *BRAF^V600E^* is involved in dedifferentiation, genomic instability as well as increased invasiveness of cancer [26]. Numerous studies show that *BRAF* mutation correlates with advanced disease, the incidence in older age, classical papillary as well as poor prognosis and poorer overall survival [27].

Around 11% of PTCs are found to have *RAS* gene family mutations (0%–11%) [28]. The highest incidence is found in the follicular variant of PTC, 43%. Mutations in the *RAS* gene generally affect codon 61 of *H-RAS* and *N-RAS* and, less often, codons 12 and 13. Mutations in the other codons and in the *K-RAS* gene are rare [28]. Tumors, which harbor the *RAS* mutation, are invariably found encapsulated, have a follicular morphology, and show lower rates of nodal disease resulting in a more favorable prognosis. Furthermore, other studies have shown a high rate of *RAS* mutations in benign tumors, e.g., up to 50% micro follicular adenomas possess *RAS* mutations. This suggests that these genetic mutations may be the result of an early event in follicular thyroid tumorigenesis [29]. Additionally, *RAS* mutations are also found in about 50% of poorly differentiated and ATCs and these mutations correlate with poor patient survival [30]. This is highly indicative of the distinct roles that RAS may play in the early and late stages of thyroid cancer.

In one of our earlier studies [31], the above-mentioned genetic alterations were detected in 150 of 266 Serbian PTC patients (56.4%). *BRAF^V600E^* was the most abundant mutation noted (84/266, 31.6%). *RET/PTC* rearrangements were found in 55/266 (20.7%) cases, the *RAS* mutations were the least frequently seen (11/ 266, 4.1%). We concluded that following radical thyroid surgery followed by radioiodine ablation, *BRAF^V600E^* may not be an appropriate measure of poor disease-free survival during the early and middle follow-up period [31].

Other genetic alterations have been identified in PTC, such as *PTEN* and *PIK3CA* mutations [7]. However, their prevalence of approximately 1%–2% and lack of specificity limit their biomarker potential in PTC. In the past decade, a significant number of studies were focused on telomerase reverse transcriptase (*TERT*) promoter mutations in thyroid cancer, as reviewed in [32]. Two *TERT* promoter mutations, C228T and C250T, have been identified, having a prevalence of 11.3% in PTC. They have been found to be associated with aggressive PTC features, tumor recurrence, and patient mortality. Moreover, in coexistence with *BRAF^V600E^*, they show a strong synergistic effect on PTC aggressiveness [32].

A number of gene expression profiles have been identified and proposed for the prediction/prognosis of PTC by various studies [33–36]. However, this is an evolving field and these results need to be reproduced and confirmed by other studies in order to pave their way to clinical practice.

Aside from the gene expression at the mRNA level, expression alterations at the protein level might also have a significant biomarker potential in PTC. A recent systematic review and meta-analysis of the programmed death-ligand 1 (PD-L1) expression level in thyroid carcinoma pointed to the PD-L1 protein expression as a potential biomarker of disease-recurrence in patients with PTC [37].

MicroRNA expression profiles have also been the focus of a plethora of studies investigating their potential as diagnostic/prognostic/predictive biomarkers in PTC and a great number of microRNAs have been found to have deregulated expression [38, 39]. A meta-analysis, including 15 studies involving 807 PTC patients, found that expression levels of miRs-21, -34b, -130b, -135b, -146b, -151, -181b, -199b-5p, -221, -222, -451, -623, -1271, -2861, and let-7e showed significant association with at least one aggressive feature, such as large tumor size, extrathyroidal extension, multifocality, vascular invasion, lymph node metastases, distant metastasis, advanced TNM stage, and presence of the BRAF(V600E) mutation [40]. According to several reports, PTC is most consistently associated with the overexpression of miR-146b, miR-221, and miR-222. Considering that overexpression of these three microRNAs is frequently associated with more aggressive PTC features, their expression profile has been proposed as a potential prognostic biomarker of PTC [39, 41, 42].

## VHL tumor suppressor

### *VHL* gene

**Figure 1.**
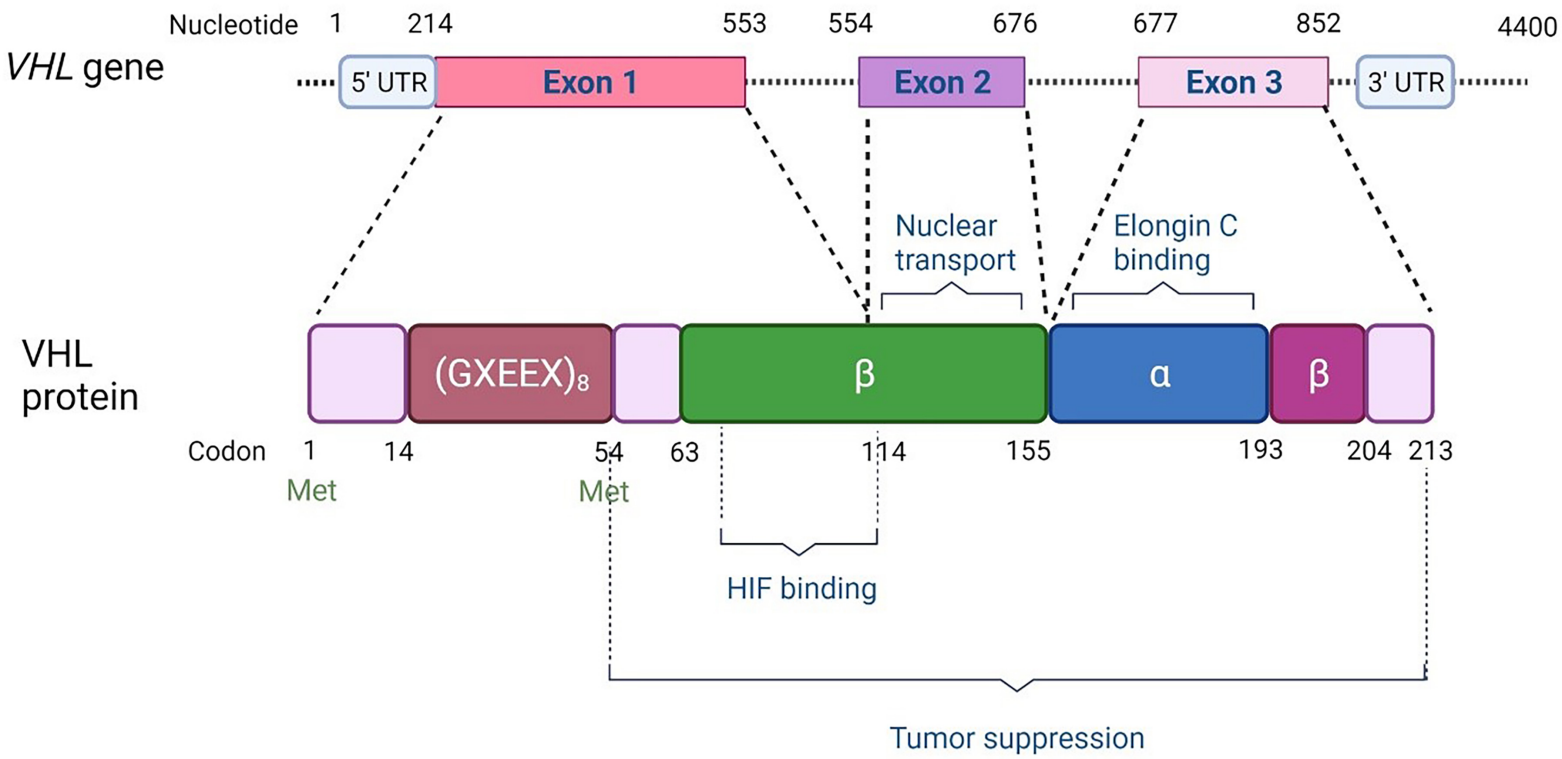
***VHL* gene and protein structure.** The figure is modified under CC BY, based on [133].

The *VHL* gene, located on chromosome 3p25, is 10 kb and comprises of three exons (Figure 1). Distinct isoforms, derived from alternative spliced transcripts have been observed. The best studied is transcript variant 1, which contains all three exons and results in two translation products: a 28- to 30-kDa 213 amino acid protein (pVHL30) translated from the first methionine codon and an 18- to 19-kDA 160 amino acid protein (pVHL19), translated from the second methionine at codon 54. In comparison to pVHL30, the first 53 amino acids are absent from pVHL19 and are less evolutionarily conserved than the rest of the protein [17]. The functional significance of this region is unclear. Both pVHL19 and pVHL30 are biologically active, have equivalent effects in functional assays, and display tumor suppressor activity in *in vivo* assays [43–47].

VHL disease is a cancer syndrome which is inherited in a dominant manner and its development predisposes to a number of other cancers linked to mutations in the *VHL* gene. The disease shows marked variation in expression with multifocal and highly vascularized tumors in both mesenchymal and neural crest-derived tissues of multiple organ systems, such as the endocrine system (islet cell tumor), central nervous system (haemangioblastoma—CNS HB), adrenal medulla (pheochromocytoma—PHE), eye (retinal haemangioblastoma—RB), and kidney (clear renal cell carcinoma—cRCC) [17, 18]. Most of the VHL disease cases examined have been shown to exhibit autosomal inherited germline mutations in the *VHL* gene with over 1000 germline and somatic mutations reported [48]. Within the characterized gene alterations, missense mutations account for approximately 52%, frameshift and nonsense mutations account for 13%  and 11%, respectively, inframe indels for about 6%, and deletion of the whole gene for accounts for about 11%. These alterations can be found throughout the coding sequence [48, 49]. Sporadic RCC and CNS have been reported to exhibit somatic mutations in the *VHL* gene [50] while in other sporadic cancers, such as breast, colon, lung, prostate, and thyroid, they are very rare [51, 52].

### VHL protein function and its role in tumor suppression

**Figure 2.**
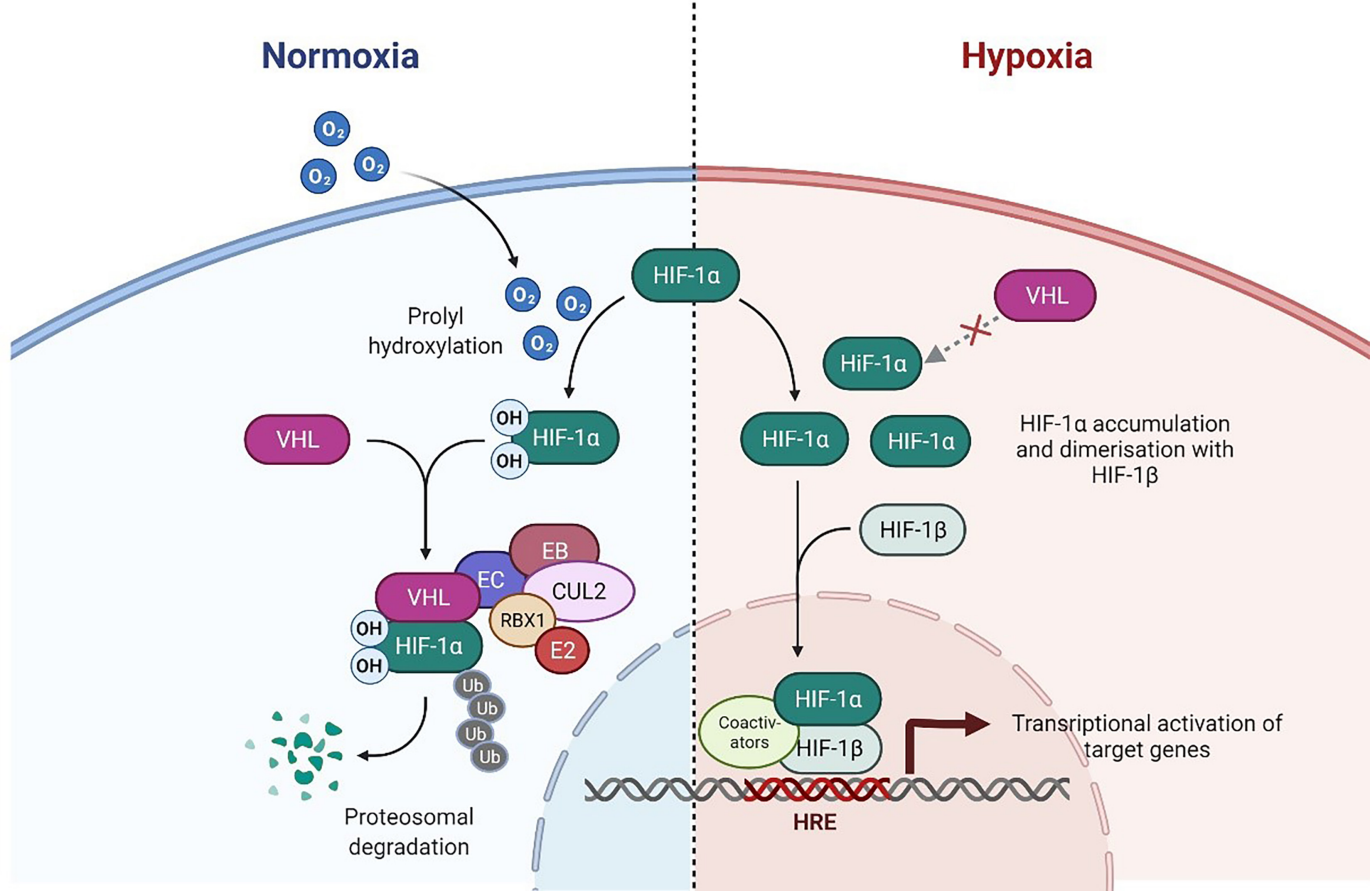
**Regulation of the HIF-α by E3 ubiquitin ligase complex in normoxic and hypoxic conditions.** Adapted from “HIF Signaling,” by BioRender.com (2021). Retrieved from https://app.biorender.com/biorender-templates.

The VHL protein forms a part of a multiprotein complex. This complex has E3 ubiquitin ligase activity that results in polyubiquitination and proteosomal degradation of particular target proteins. Other members of this complex are elongin B, elongin C, cullin-2 (CUL2), and RING-box1 (RBX1). The main role of VHL in the complex is recognition of the specific protein targets, which are then marked for degradation [53–55]. One particular protein target of VHL, the hypoxia-inducible factor-a (HIF-α), has been the focus of many studies. HIF-α is a transcription factor which plays a pivotal role in the regulation of gene expression by oxygen [55–59] (Figure 2).

HIF-α is recognized and marked for degradation under normoxia conditions. In cells exposed to low oxygen levels or which lack functional VHL, HIF-α subunits accumulate and complex with the HIF-β subunit, forming heterodimers. Formation of this heterodimer results in the activation of a number of genes leading to the production of proteins involved in cell adaptation to hypoxia and regulation of angiogenesis [55, 59, 60].

Accumulating evidence suggests that the function of VHL is broader than its established role in oxygen signal transduction. Moreover, the loss of VHL function may affect the regulation of other signaling pathways with important roles in biological processes, such as cell survival, invasion, proliferation, and metastasis [19, 20, 61]. It was found that the VHL protein interacts with a variety of other proteins in the cell, leading to their degradation or inhibition. For example, subsequent to VHL protein interaction, the HIF deubiquitinating enzymes VDU1/2 [62, 63] and Rpb1 subunit of RNA polymerase II are marked for degradation [64]. Studies show that VHL can also inhibit activity in several members of the protein kinase C family [65–67] and the activity of the Sp1 transcription factor [68, 69]. Furthermore, VHL was demonstrated to interact with the ubiquitously expressed Hu family RNA-binding protein HuR [70] that plays a part in mRNA stabilization and, with fibronectin, contributing to the proper assembly of the extracellular matrix [71, 72]. VHL was also found to interact with microtubules and protect them from depolymerization [73]. According to some studies, VHL acts as a positive regulator of the tumor suppressor TP53 (tumor protein p53) by inhibiting its Mdm2-mediated ubiquitination, and by subsequent recruitment of p53-modifying enzymes [74, 75]. On the other hand, there is evidence that VHL negatively regulates p53 activity by controlling the formation of p53 tetramers and reducing the binding of p53 at the promoters of the target genes [76]. A number of other VHL substrates/binding partners and associated signaling pathways have recently been identified, as extensively reviewed elsewhere [61].

There is mounting evidence that VHL performs a wide variety of HIF-α-dependent as well as HIF-α-independent functions affecting thus different cellular processes, some of which have a crucial role in tumorigenesis [50, 77]. It is still unclear, however, to which extent these HIF-α-dependent and HIF-α-independent functions cooperate during the process of tumorigenesis. A summary of the VHL protein functions and their associations with various processes implicated in tumor pathology is given in Tables 1A and 1B.

**Table 1A:** HIF-α-dependent functions of VHL protein and their association with cellular processes involved in tumor development and progression. The table is based on data from [50, 55, 61, 118–120]

**Process**	**HIF-α-dependent functions**
Cell proliferation and survival	Regulation of TGFα and EGFR
Apoptosis	HIF modulation of p53and NF-κB activity, and suppression of BNIP3
Cell cycle progression	Regulation of cyclin D1
Angiogenesis	Regulation of VEGF, PDGF among others
Glucose uptake and metabolism	Regulation of GLUT1, GLUT3, HK2, PGK1, LDHA, PFK, and PDH, among others
Microtubule stabilization and maintenance of the primary cilium	Primary cilia modulation
Chemotaxis	Regulation of SDF1 and CXCR4
Assembly and regulation of the extracellular matrix	Regulation of E-cadherin and MMPs
Homeostasis	Regulation of external pH through CAIX

HIF: Hypoxia-inducible factor; TGFα: Transforming growth factor-α; EGFR: Epidermal growth factor receptor; NF-κB: Nuclear factor-κB; BNIP3: BCL2/adenovirus E1B-interacting protein 3; VEGF: Vascular endothelial growth factor; PDGF: Platelet-derived growth factor; GLUT: Glucose transporter; HK2: Hexokinase 2, PGK1: Phosphoglyceratekinase 1; LDHA: Lactate dehydrogenase A; PFK: Phosphofructokinase; PDH: Pyruvate dehydrogenase; SDF1: Stromal-cell derived factor 1 (encoded by *CXCL12*); CXCR4: CXC-chemokine receptor 4; MMPs: Matrix metalloproteinases; CAIX: Carbonic anhydrase IX.

**Table 1B:** HIF-α-independent functions of VHL protein and their association with cellular processes involved in tumor development and progression. The table is based on data from [50, 55, 61, 118–120]

**Process**	**HIF-α-independent functions**
Cell proliferation and survival	Regulation of NDRG3, which accumulates by binding to lactate under hypoxia and further interacts with c-Raf for the activation of the Raf-ERK pathway. Regulation of AKT–VHL binds to hydroxylated AKT induced by EglN1 and inhibits its phosphorylation and kinase activity.
Apoptosis	Activation of p53 transcriptional activity, modulation of NF-κB activity and downregulation of JUNB (which is known to blunt neuronal apoptosis during NGF withdrawal).
Cell cycle progression	VHL targets B-Myb (MYBL2) for ubiquitination and proteasome degradation
Cell senescence	Control of cell senescence through RB and the SWI2/SNF2 chromatin remodeller p400
Transcriptional regulation	Involvement in ubiquitylation of the large subunit of RNA polymerase II in response to oxidative stress, control of influence on HuR, binding to SP1 transcription factor
Microtubule stabilization and maintenance of the primary cilium	Association and stabilization of microtubules. Ubiquitination of Aurora kinase A (AURKA) independent of oxygen-dependent PHD activity to regulate formation of the primary cilium in quiescent cells
Assembly and regulation of the extracellular matrix	Regulation of fibronectin, collagen IV, adherens, tight junction, integrins and MMPs
Homeostasis	Control of cell senescence through RB and the SWI2/SNF2chromatin remodeller p400
Inflammation	VHL functions as an adaptor that promotes the inhibitory phosphorylation of the NF-κB agonist, Card9, by enhancing the interaction between Card9 and CK2
Cell growth and proliferation	Interacts with RAPTOR and increases RAPTOR degradation by ubiquitination, thereby inhibiting mTORC1 signaling
Cell growth, apoptosis, cell differentiation, stem-cell self-renewal	Negative regulation of c-Myc transcription
Anthracycline cytotoxicity regulation	Transcritional regulation of *ALDH2* through interaction with its transcription factor HNF-4α

NDRG3: N-Myc downstream-regulated gene 3; NF-κB: Nuclear factor-κB; HIF: Hypoxia-inducible factor; HuR: Human antigen R (also known as ELAV1); NGF: Nerve growth factor; RB: Retinoblastoma protein; CARD9: Caspase Recruitment Domain Family Member 9; CK2: Casein kinase II; RAPTOR: Regulatory-associated protein of the mechanistic target of rapamycin complex 1 (mTORC1); ALDH2: Aldehyde dehydrogenase 2; HNF-4α: Hepatocyte nuclear factor 4 alpha.

### VHL expression in PTC

VHL has been shown to be aberrantly expressed in a number of human cancers. These include kidney, colon, breast, gastric cancer, and MEN2-associated medullary thyroid cancer [51, 78, 79]. A few studies have investigated the potential involvement of VHL in PTC development and/or progression. The VHL protein is highly expressed in normal thyroid follicular tissue and is differentially expressed in non-neoplastic and neoplastic thyroid lesions in proportion to the level of tumor differentiation [80–82]. This led to our hypothesis that VHL may be involved in the development of PTC. Consequently, we conducted a study evaluating mutation and methylation status as well as levels of expression of the *VHL* gene in tumour samples from 264 patients presenting with PTC. We found no somatic mutations or evidence of *VHL* downregulation via promoter hypermethylation. However, we found strong evidence of deregulated *VHL* expression at the mRNA level. Moreover, low *VHL* mRNA levels showed a strong correlation with patients’ older age, advanced clinical stage of the disease, classical PTC histovariant, and tumor multifocality. We also detected a marginal influence of low *VHL* expression on disease-free interval [83]. Our study was the first to demonstrate the association between *VHL* levels and clinico-pathological parameters in PTC, providing evidence of the involvement of VHL tumor suppressor in PTC pathology.

Later, in a similar study, Baldini et al. measured the expression levels of the two *VHL* mRNA splicing variants, VHL-213 (V1) and VHL-172 (V2), in a series of 96 PTC and corresponding normal thyroid tissues. They reported that expression of *VHL* was deregulated in most of the PTC tissues analyzed, and that the percent of samples with downregulated expression levels of both splicing variants was slightly higher than the percent of samples with upregulated V1 and V2 expression levels [84]. The mechanisms responsible for *VHL* gene expression regulation were not investigated in this study*.* In our second study on *VHL* in PTC [85], we compared the expression levels of *VHL* mRNA in another tumor series consisting of 42 pairs of PTCs and matched non-tumor thyroid tissues. The results showed that compared to corresponding non-tumor thyroid tissues, the levels of *VHL* in tumor tissues were either up- or downregulated, which was in line with the results of Baldini et al. [84], despite the opposite trend in the percent of the decreased and increased cases in these two studies. We also evaluated the association between *VHL* expression levels and clinico-pathological parameters in this patient cohort. Our data showed that lower *VHL* levels were significantly associated with extrathyroid spread and capsular invasion and there was a trend toward association with the presence of lymph node metastases, which led to the overall conclusion, consistent with our first study of VHL in PTC, that VHL downregulation might be associated with more aggressive tumor features, at least in some PTC cases.

Later on, two other studies addressed the status of VHL in PTC. Zang et al. [86], who evaluated the *VHL* expression in PTC and corresponding normal thyroid tissues in a group of 52 PTC patients, reported that *VHL* levels were significantly decreased in PTC. Deregulated *VHL* gene expression was also found in a recent study conducted on 20 primary tumor and metastatic PTC tissue. Interestingly, lower *VHL* mRNA levels were found in primary tumors compared to metastatic tissues. In primary tumors, BRAF^V600E^ positive status was associated with higher levels of *VHL*, while in metastatic tissue, it was associated with lower *VHL* levels [87].

Summarized results produced from other gene profiling studies showed differences in the expression of over 200 other genes shared between PTC and normal thyroid tissues. The upregulated expression of *LGALS3*, *SERPINA1*, *MET, KRT19*, *FN1,* and *TIMP1* was found within the existing data, as well as downregulated expression of *TPO*, *SLC26A4*, *DIO1/2,* and *TFF3* in the well differentiated thyroid carcinomas but there was no evidence of deregulated expression of *VHL* [42]. This could be attributed to the small sample size of most of the studies or the cut-off values for differential expression being set too high. On the other hand, *VHL* has been reported to be included in a robust predictive signature for patients with breast cancer. Based on RNA-seq data from The Cancer Genome Atlas and several Gene Expression Omnibus datasets, a 14-gene hypoxia-related signature, which included *VHL*, was developed and the findings revealed that this signature could serve as a potential prognostic biomarker for breast cancer [88]. 

### VHL expression in other types of cancer

Looking at existing data on the expression of VHL and its correlation with clinicopathological features in other cancer types, Zia et al. [89] reported that in highly aggressive breast cancer cell lines, VHL was either not expressed or was expressed at a low level, affecting cell motility and invasiveness. Zia et al. also found that, in higher grade breast cancer tumors, VHL was expressed at a much lower level compared to its expression in lower grade breast cancer tumors. The downregulated expression of VHL was also seen in tumors from patients with nodal and distant metastasis [89]. A study on ovarian cancer cells also showed that the loss of VHL increased cell aggressiveness [90]. Reduced pVHL expression has also been shown to be correlated with decreased apoptosis and a higher grade of chondrosarcoma [91]. Hoebeeck et al. [92] report that neuroblastoma patients also show a strong correlation between reduced levels of  VHL and a poorer outcome in terms of patients’ survival. Similarly, in clear cell renal cell carcinoma, the increase in tumor aggressiveness was found to correlate with reduced expression of VHL identifying VHL downregulation as a risk factor for worse patient overall survival [93]. According to a study of Li et al. [94], although no correlations were observed with patient age, sex, tumor size, lymph node metastasis, or distant metastasis, negative VHL expression associated with a worse prognosis in patients with hepatocellular carcinoma. In a recent study on bladder cancer, differential under-expression of VHL—both mRNA and protein—was found in muscle-invasive bladder cancer in comparison to non-muscle-invasive bladder cancer [95].

### Major mechanisms of *VHL* gene inactivation in cancer

Inactivation of the *VHL* gene can result from various alterations, such as intragenic mutations, mitotic recombination events, and promoter hypermethylation. *VHL* gene mutations were found in tumors associated with VHL syndrome as well in some sporadic tumors, such as clear-cell renal carcinomas, hemangioblastomas, and sporadic pheochromocytomaarise harbor *VHL* gene mutations [96–98]. Somatic *VHL* mutations on the other hand are rare in histological tumor types not present in VHL disease [51]. The results of our study, which found no evidence for mutations or homozygous deletions of the *VHL* are consistent with these reports [83]. However, loss of heterozygosity at chromosome 3p, including the *VHL* gene locus (3p25), was reported in one study [99] where it was found in 29% of PTCs.

The other common mechanism of gene inactivation is the hypermethylation of the promoter region. The *VHL* gene has been found to be silenced by methylation in 20%–30% of individuals with renal cell carcinoma, acute myeloid leukemia, or multiple myeloma [100–102] while in plasma cell neoplasia methylation of the *VHL* promoter is a common event [103]. In a recent study on bladder cancer, promoter methylation of the *VHL* gene was detected in almost 43% of bladder cancer samples, with high methylation being more frequent in muscle-invasive bladder cancer than in non-muscle-invasive bladder cancer [95]. Methylation of the *VHL* promoter was also detected in different stages of cervical carcinoma [104]. Several groups have reported the presence of epigenetic modifications in thyroid. Promoter hypermethylation was detected in the following tumor suppressors: *CDH1, p16INK4A, RASSF1A*, *SLC5A8, TIMP3, DAPK, MGMT, DNMT1, MLH1,* and *RARB* among others [105–110]. The methylation status of *VHL* in PTC patients has so far been addressed by only a couple of studies. Migdalska-Sk et al. [111] analyzed the methylation levels of eight tumor suppressor genes, including *VHL*, in PTC and control, non-cancerous thyroid tissues. According to this study, the highest methylation rate—100%, was found in *ARHI, CDH1, p16INK4A,* and *RASSF1A* but the frequency of promoter methylation of the *VHL* gene was the lowest, both in PTC and noncancerous tissues [111]. Similarly, the analysis of our PTC sample series with reduced *VHL* levels did not find evidence for *VHL* gene silencing through methylation. However, since our analysis covered only one part of the *VHL* promoter we could eliminate the possibility of the presence of methylation in the promoter regions that were not analyzed in our studies [83].

Small non-coding RNAs (microRNAs, miRNAs) have a significant role in gene expression downregulation [112]. This is a class of ~22 nucleotides long non-coding RNAs involved in the posttranscriptional regulation of gene expression. They typically bind to the 3 untranslated regions (UTRs) of target gene mRNAs, which leads to degradation or to translation inhibition of the target mRNA, resulting in expression downregulation of their protein products [113]. Since their discovery, a plethora of studies have demonstrated the importance of miRNAs in cancer biology, with their activity being shown to affect a number of crucial processes in tumorigenesis, such as tumor growth, invasion, angiogenesis, and immune evasion. Depending on their targets, miRNAs can function as oncogenes or tumor suppressors [114]. A number of miRNAs were reported to target *VHL* directly, downregulating its expression in different cancers, as summarised in Table 2.

**Table 2:** MicroRNAs experimentally confirmed to regulate *VHL* expression in different types of cancer cells

**microRNA**	**Type of cancer cells**	**Reference**
miR-17-5p	Renal cell carcinoma cells	[121]
miR-21	Hepatic stellate cells; papillary thyroid carcinoma; pancreatic carcinoma; cervical carcinoma cells	[86, 122–124]
miR-23b	Glioma cells	[125]
miR-92	Epithelial ovarian carcinoma; clear cell renal cell carcinoma	[116, 117]
miR-101	Breast carcinoma cells	[126]
miR-150	Glioma cells	[127]
miR-155	Breast carcinoma cells	[78]
miR-222	Retinoblastoma cells	[128]
miR-224	Renal cell carcinoma cells	[121]
miR-331-3p	Hepatocellular carcinoma cells	[129]
miR-429	HER2+ breast carcinoma cells	[130]
miR-566	Glioblastoma cells	[131]
miR-887	Hepatocellular carcinoma cells	[132]

So far, few studies have addressed the regulation of *VHL* by miRNAs in PTC. Zang et al. [86] showed that *VHL* can be a potential target of miR-21 in PTC cells. MiR-21 is an oncomiR involved in the tumorogenisis of a number of different cancers [115] and, according to several reports, as summarized in Table 2, it can directly target *VHL* in different cancers. In one of our studies, we measured the expression levels of *VHL* and another well documented oncomiR—miR-92a-3p—and explored the correlation between them in PTC and nontumor thyroid tissue. We found that both *VHL* and miR-92a were deregulated in PTC but a negative correlation between them existed only in a subgroup of PTCs with vascular invasion. Based on these results, we can speculate that *VHL,* at least at some points during tumor progression, might be regulated by miR-92a-3p in PTC as well, since the possibility of their direct interaction was demonstrated in renal cell carcinoma and epithelial ovarian carcinoma cells [116, 117]. However, more research needs to be done in order to discover the complex interaction network between VHL and functionally related miRNAs in different stages of PTC development and progression, as well as to clarify their roles in disease progression and their prognostic utility.

## Conclusion

The VHL tumor suppressor has been implicated in the development of a dominantly inherited cancer syndrome known as VHL disease, as well as a number of sporadic cancers. By regulation of the availability of HIF-α in the cell, the VHL has important effects on the tumorigenesis of the cell. VHL protein negatively controls angiogenesis, a critical factor in the progression of cancer. Accumulating evidence strongly indicates that VHL is also involved both through HIF-α-dependent as well as HIF-α-independent actions in several other processes, such as cell proliferation and survival, cell cycle progression, apoptosis, extracellular matrix regulation, inflammation, etc. Moreover, the latest evidence suggests that aside from a tumor suppressor function, VHL may also demonstrate pro-tumor function in some circumstances. In this context, VHL has definitively been shown to be a strong potential candidate as a biomarker and/or a therapeutic target in cancer. However, more research needs to be done since the complexity of its role in the cell, both in normal and pathological conditions, has only recently started to be revealed. So far, just a few studies have investigated VHL in papillary thyroid cancer, and all reported it to be deregulated. The significance of this deregulation, as well as its potential as a diagnostic/prognostic biomarker has yet to be clarified. In this review, we summarized the existing knowledge about the implication of VHL in PTC pathogenesis with the aim to bring attention to it and emphasize its potential utility as an expression biomarker for the stratification of PTC patients into high and low risk groups for recurrent disease.

## Acknowledgments

Figures in this article were created with BioRender.com

**Conflicts of interest:** The authors declare no conflicts of interest.

**Funding:** This work was funded by the Ministry of Education, Science and Technological Development of The Republic of Serbia, grant number 451-03-9/2021-14/ 200017 and Cell and Gene Therapy group, King’s College London, The Rayne Institute, 123 Cold harbour Lane, London SE59NU, UK.
